# An Exogenous Surfactant-Producing *Bacillus subtilis* Facilitates Indigenous Microbial Enhanced Oil Recovery

**DOI:** 10.3389/fmicb.2016.00186

**Published:** 2016-02-18

**Authors:** Peike Gao, Guoqiang Li, Yanshu Li, Yan Li, Huimei Tian, Yansen Wang, Jiefang Zhou, Ting Ma

**Affiliations:** Key Laboratory of Molecular Microbiology and Technology, Ministry of Education, College of Life Sciences, Nankai UniversityTianjin, China

**Keywords:** microbial enhanced oil recovery (MEOR), *Bacillus subtilis*, surfactants, microbial community, stimulation, high-throughput sequencing

## Abstract

This study used an exogenous lipopeptide-producing *Bacillus subtilis* to strengthen the indigenous microbial enhanced oil recovery (IMEOR) process in a water-flooded reservoir in the laboratory. The microbial processes and driving mechanisms were investigated in terms of the changes in oil properties and the interplay between the exogenous *B. subtilis* and indigenous microbial populations. The exogenous *B. subtilis* is a lipopeptide producer, with a short growth cycle and no oil-degrading ability. The *B. subtilis* facilitates the IMEOR process through improving oil emulsification and accelerating microbial growth with oil as the carbon source. Microbial community studies using quantitative PCR and high-throughput sequencing revealed that the exogenous *B. subtilis* could live together with reservoir microbial populations, and did not exert an observable inhibitory effect on the indigenous microbial populations during nutrient stimulation. Core-flooding tests showed that the combined exogenous and indigenous microbial flooding increased oil displacement efficiency by 16.71%, compared with 7.59% in the control where only nutrients were added, demonstrating the application potential in enhanced oil recovery in water-flooded reservoirs, in particular, for reservoirs where IMEOR treatment cannot effectively improve oil recovery.

## Introduction

With the increasing global energy demand and depletion of oil reserves, oil recovery by microbial flooding is currently under intensive development, and has been shown to be economically feasible by laboratory and field trials (Belyaev et al., 1998; Gao and Zekri, 2011; Xiao et al., 2013; Li et al., 2014; Shibulal et al., 2014; Dong et al., 2015; You et al., 2015). This technique is generally classified into exogenous and indigenous microbial enhanced oil recovery (MEOR). Exogenous MEOR includes injection of exogenous microorganisms and *ex situ*-produced products into reservoirs to enhance oil recovery (Zobell, 1947). This is an effective way to quickly establish the appropriate activity in reservoirs (Youssef et al., 2009, 2013). The drawback is that these microorganisms must be able to grow in the presence of competing indigenous populations (Bryant, 1991). Furthermore, because of the sieve effect of strata on microbial cells, the injected microorganisms are generally difficult to migrate into reservoir strata to reach the production wells (Youssef et al., 2009; Gao et al., 2015a; Ren et al., 2015). Indigenous microbial flooding is another promising oil-recovery technique that has been successfully applied in the petroleum industry (Belyaev et al., 1998; Liu et al., 2005; Li et al., 2014; Wang et al., 2014b; Le et al., 2015). This technique improves oil recovery by stimulating reservoir microorganisms through introducing air and nutrients into reservoir strata. However, this technique generally needs long incubation times before indigenous microorganisms grow in large numbers and produce sufficient metabolites, which means that the limited air supply will be exhausted by saprophytic bacteria, leading to an inefficient surfactant-producing process.

**FIGURE 1:**
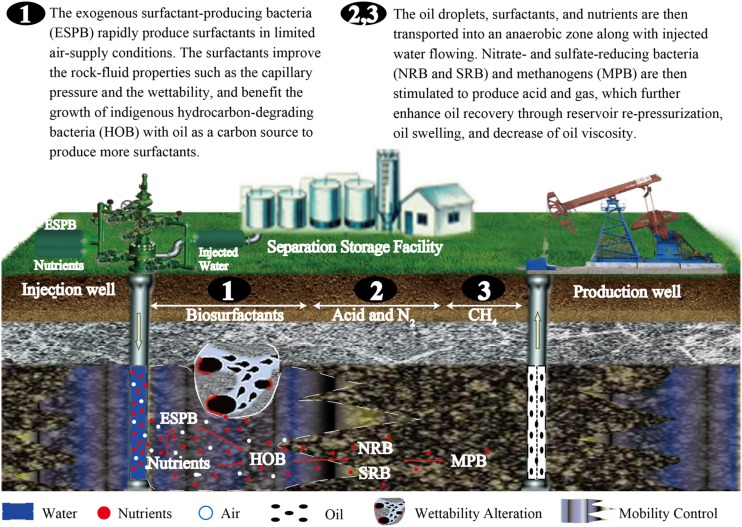
**Schematic showing how exogenous surfactant-producing bacteria facilitate indigenous microbial enhanced oil recovery**.

Surfactant production is thought to be the initial critical stage of indigenous microbial flooding, and is beneficial for the stimulation of other anaerobic populations that follow in the reservoir ecosystem (**Figure 1**). Thus, adding exogenous surfactant-producing bacteria may be an appropriate way to improve this process during indigenous microbial flooding. The injected exogenous bacteria will grow and colonize the aerobic zone of injection wells, and rapidly produce larger amounts of surfactants, which not only reduces the interfacial tension between brines and the oil phase, but also emulsifies oil into droplets. The oil droplets further enhance the growth of indigenous hydrocarbon-degrading bacteria with oil as the carbon source to produce more surfactants. The partially emulsified oil, surface-active agents, and nutrients are then transported into the reservoir strata (an anaerobic zone) along with injected water. Then, nitrate- and sulfate-reducing bacteria and methanogens are stimulated to produce acid, alcohol, and gas, which further enhances oil recovery through reservoir re-pressurization, oil swelling, and decrease of oil viscosity.

To date, several surfactant-producing populations, in particular *Pseudomonas* and *Bacillus*, have been injected into reservoir strata to improve oil production (Simpson et al., 2011; Youssef et al., 2013; You et al., 2015). These studies also showed the feasibility of *in situ* biosurfactant production and its potential to improve oil production. However, the microbiological processes that take place in the MEOR process have not yet been elucidated. How do the injected microbial populations strengthen and improve the indigenous microbial flooding process? What is the interplay process taking place between the injected microbial populations and the indigenous microorganisms? To explore these issues, we used an exogenous *Bacillus subtilis* strain M15-10-1 to strengthen the indigenous microbial enhanced oil recovery process. The microbial processes and driving mechanisms were investigated by analyzing the changes in oil properties and the interplay between the exogenous *B. subtilis* and indigenous microbial populations in microcosms stimulated with different kinds of nutrients. The application potential for enhancing oil recovery was verified by a core oil-displacement test.

## Materials and Methods

### Water Samples Collection and Nutrients Selection

Water samples were taken through sampling valves located at the wellhead of production wells in the Lu water-flooded petroleum reservoir in Xinjiang Oil Field, China. This reservoir is a sandstone reservoir that has been subjected to water flooding since 2001. The depth of the oil-bearing strata is less than 1200 m with a temperature of 37°C and a formation pressure of 10.2 MPa. The porosity of the reservoir is 29.9%, with an average permeability of 522 × 10^–3^ μm^2^. The viscosity of the crude oil is 18 mPa-s with a density of 0.846 g cm^–3^. The physicochemical characteristics of the formation brines indicated extremely low levels of phosphate and nitrate sources (Supplementary Table S1). Based on the nutrient deficiency in the formation brines, nutrient compounds containing carbon, nitrogen, and phosphorus were selected for the following microbial stimulation.

### Strain M15-10-1 and its Metabolic Characteristics

Strain M15-10-1 was isolated from the produced water of a mesothermal water-flooded petroleum reservoir. The 16S rRNA sequence and the lipopeptide-producing gene *srfA* were amplified using the primer sets 27F-1541R and srfAF-srfAR (**Table 1**). The obtained sequences were aligned with related species on the National Center for Biotechnology Information (NCBI) database, and were used to construct phylogenetic trees based on the neighbor-joining method with 1000 bootstrap replicates using MEGA 4 (Tamura et al., 2007). To test the ability of the strain to grow and produce surfactants on various carbon sources, water-soluble substrates and water-insoluble carbon sources, including glucose, sucrose, molasses, corn steep powder, ethanol, glycerol, sodium acetate (NaAc), peanut oil, bean oil, and hydrocarbons, were investigated. Each substrate was sterilized by filtration through a 0.45 μm filter before being added to heat-sterilized basic salts medium (BSM) for fermentation, which was performed at 40°C in a 250 mL flask containing 50 mL fermentation medium. BSM included the following nutrients: 10 g L^–1^ NaH_2_PO_4_⋅2H_2_O, 2 g L^–1^ K_2_HPO_4_⋅3H_2_O, 2 g L^–1^ NH_4_NO_3_, 0.2 g L^–1^ MgSO_4_⋅7H_2_O, and 0.01 g L^–1^ yeast extract powder, with a pH of 7.2.

**Table 1:** Primers used in the present work for qPCR.

Target	Primer set	Sequence	PCR conditions
*Bacillus subtilis*	srfAF	5-CAAAATCGCAGCATACCACTTTGAG-3	56° C
M15-10-1	srfAR	5-AGCGGCACATATTGATGCGGCTC-3	30 cycles
*Dietzia* sp. ZQ-4	algF	5-GTCCACCACGAAGCAGC-3	56° C
	algR	5-CCTACAACGGCATCAAACTG-3	30 cycles
*Rhodococcus* sp. M	alkF	5-AGTGGGCGCTCGCCCCGTCGTTCTAC-3	56° C
	alkR	5-CACGAGATACGGCGCGATCGACAGAC-3	30 cycles
*Enterobacter cloacae*	895F	5-GGCAGCGTGTCAAACTCAA-3	57° C
FY-07	895R	5-TTTACCGACGGCTCACAGAT-3	30 cycles
*Enterobacter cloacae*	895F	5-GGCAGCGTGTCAAACTCAA-3	57° C
Bacteria	27F	5-AGAGTTTGATCCTGGCTCAG-3	55° Cs
	1541R	5-AAGGAGGTGATCCAGCCGCA-3	30 cycles
Bacteria	8F	5-AGAGTTTGATYMTGGCTC-3	55° C
	338R	5-GCTGCCTCCCGTAGGAGT-3	30 cycles
Hydrocarbon degrader	alkBwF	5-AAYCANGCNCAYGARCTNGGVCAYAA-3	55° C
	alkBwR	5-GCRTGRTGRTCHGARTGNCGYTG-3	30 cycles

Microbial growth curves were measured by determining the optical density of the culture (OD_600_). The surface tension was measured at room temperature using a digital tension meter (POWEREACH JK99B, China). The crude surfactant was separated from the culture medium using acid precipitation and the solvent extraction method (Liu et al., 2012). The collected brownish oily residue was ground with KBr powder, dispersed uniformly in a matrix of paraffin, and compressed to form an almost transparent disk for Fourier transform infrared spectrometry (FTIR) measurement in the frequency range of 4,000–500 cm^–1^. The oil-degrading ability of the *B. subtilis* was investigated in a 250 mL flask containing 100 mL BSM with 2% crude oil. Cultivation was carried out at 40°C under aerobic conditions. The oil degradation was analyzed according to the following methods.

### Growth of *Bacillus subtilis* M15-10-1 in an Artificial Microflora

To investigate the inhibitory effect of the *B. subtilis* on reservoir microbial populations, a microflora, including the *B. subtilis* M15-10-1, *Dietzia* sp. ZQ-4, *Rhodococcus* sp. M, and *Enterobacter cloacae* FY-07, was constructed. The strains were all isolated from oilfield environments. The four strains were first inoculated into 5 mL LB culture medium, and shaken overnight at 40°C on a rotary shaker at 180 rpm. Then, 10 μL cultures were inoculated into a 250 mL flask containing 100 mL LB or 100 mL BSM with 2% crude oil, and were shaken at 180 rpm at 40°C. The numbers of the four stains were determined at different time points by quantitative PCR (qPCR). The molecular markers and PCR conditions used to quantify the four stains are listed in **Table 1**. The reaction systems were denatured for 2 min at 95°C followed by 35 cycles at 94°C for 30 s, annealing for 30 s, and 72°C for 30 s. Fluorescence was determined at the end of every 72°C extension phase in a Bio-Rad iQ5 Sequence detection system. Gene copy numbers in unknown samples were determined based on standard curves constructed from 10-fold serial dilutions of the standard as described previously (Li et al., 2014).

### Stimulation with Nutrients or Combination with *Bacillus subtilis* M15-10-1

To investigate the influence of *B. subtilis* M15-10-1 on oil properties and indigenous microorganisms, stimulation with multiple nutrients or combination with *B. subtilis* was performed in 250 mL flasks containing 100 mL of produced water derived from the reservoir. The *B. subtilis* agent was first incubated for 16 h in a 250 mL flask containing 50 mL BSM with 0.2% glucose as the carbon source to prepare the microbial agent. Then, the fermentation broth was centrifuged at 10,000 *g* for 10 min to collect the microbial cells, which were suspended with BSM in the same volume to prepare the broth-free microbial agent. Considering the nutrient deficiency in the formation brines (Supplementary Table S1), the nutrient compounds contained 0.2% (NH_4_)_2_HPO_4_, 0.4% NaNO_3_, 2% crude oil, and/or 0.2% glucose (for industrial purposes, glucose ≥ 96%), 0.2% glycerol (for industrial purposes, glycerol ≥ 95%), 0.2% molasses (sucrose ≥ 35%, reducing sugar ≥ 16%, and nitrogen compounds ≥ 4.5%), and 0.2% corn steep powder (protein ≥ 43.0%, carbohydrate ≥ 8.0%). The inoculation quantity of the broth-free *B. subtilis* agent was determined according to the microbial concentration of the produced water of the reservoir. Cultivation was carried out at 40°C under aerobic and limited air supply conditions that were realized by sealing the flasks with rubber stoppers. The oil degradation and emulsification, microbial abundance, and community compositions were analyzed according to the following methods.

### Analysis of Microbial Abundance and Community Compositions

Total genomic DNA was extracted using a bead shaker treatment and the AxyPrep™ Genomic DNA Miniprep Kit (Axygen, USA). Briefly, microbial cells were resuspended with 1 mL TE buffer (80 mM Tris, 40 mM EDTA, pH 8.0), and then lysed using a mini bead-beater (BioSpec, USA) at 200 rpm for 1 min at room temperature with 0.1 mm glass beads. After bead beating, lysozyme was added (final concentration of 1 mg mL^–1^), and the samples were incubated at 37°C for 1 h. Following the lysozyme treatment, 120 μL sodium-dodecyl sulfate (20% SDS, w/v) was added and the samples were incubated at 65°C for 60 min. Total genomic DNA was then extracted from the suspension solution using an AxyPrep™ Genomic DNA Miniprep Kit (Axygen, USA) according to the manufacturer’s instructions and finally stored at -80°C for subsequent analysis.

The universal primer set 515f (GTG CCA GCM GCC GCG GTAA) and 806r (GGA CTA CHV GGG TWT CTA AT) were used to amplify the microbial 16S rRNA gene V4 region (300–350 bp) according to the protocol described by Caporaso et al. (2011, 2012). Replicate PCR products of the same sample were mixed to remove PCR artifacts. Amplicon sequencing was conducted on an Illumina MiSeq platform at Novogene, Co., Beijing, China. Pairs of reads from the original DNA fragments were merged using fast length adjustment of short reads (FLASH; Magoc and Salzberg, 2011). Sequences were then demultiplexed and quality filtered using the default parameters of the Quantitative Insights into Microbial Ecology (QIIME) software package (Caporaso et al., 2010). The operational taxonomic unit (OTU) clustering pipeline UPARSE was used to select OTUs at 97% similarity (Edgar, 2013). The representative sequence sets were aligned and given a taxonomic classification using Ribosomal Database Project (RDP; Wang et al., 2007). The similarity among microbial communities was determined using histograms, UniFrac principal coordinates analysis (PCoA), and the unweighted pair-group method with arithmetic mean (UPGMA).

16S rRNA, and the *alkB* and *srfA* genes were used as molecular markers to quantify the total bacteria, hydrocarbon degraders, and the *B. subtilis*, respectively. qPCR of the bacterial 16S rRNA genes was performed with the primer set 8F and 338R as described by Schulz et al. (2010). Degenerate primers alkBwf and alkBwr were used to detect the *alkB* gene, which catalyzes the first step of the hydrocarbon-degradation process (Wang et al., 2010). The quantification of the *B. subtilis* was performed with the primer set srfAF and srfAR. The copy numbers and average copy numbers of the genes detected in the water samples stimulated by nutrients or in combination with *B. subtilis* M15-10-1 were calculated, and were compared using One Way Analysis of Variance (ANOVA) with Student–Newmnan–Keuls tests.

### Analysis of Oil Degradation

Residual oil was extracted using a previously described protocol (Gao et al., 2013). Briefly, the residual oil was extracted twice with chloroform, and then separated into saturated hydrocarbon, aromatic hydrocarbon, asphaltene, and non-hydrocarbon fractions using a silica gel G (60–120 mesh) column (2 cm × 30 cm). The relative quantity of saturated hydrocarbons, aromatic hydrocarbons, asphaltene, and non-hydrocarbons in crude oil was calculated. The saturated hydrocarbons were analyzed according to the Chinese Standard SY/T 5779-2008 by gas chromatography–mass spectroscopy (GC–MS) using Agilent 7890–5975 machines equipped with HP-5MS capillary columns (60 m × 0.25 mm i.d., 0.25 mm thickness).

### Core Oil-Displacement Test

The core oil-displacement test was performed to evaluate the potential application in improving oil recovery. The produced oil and water from Lu reservoir were used as the oleic and aqueous phases, respectively. The core models were 28.5–30.6 cm in length, 3.8 cm in diameter, had pore volumes of 60.3–62.1%, and water permeability of 1.97–2.15 μm^2^ (**Table 2**). The oil-saturated core models were first flooded with injection water until the water cut in the eﬄuent of the core models was higher than 98%, which means that the core reached its residual oil saturation. A total of 0.2 PV of prepared formation brine containing nutrients and 0.2 PV of an air package were then injected into the water-flooded cores. The prepared formation brine included 0.2% (NH_4_)_2_HPO_4_, 0.4% NaNO_3_, 0.2% corn steep powder and 0.5% fermentation broth of *B. subtilis* or 0.5% *B. subtilis* agent that were suspended with BSM. The *B. subtilis* inoculation quantity was selected according to the microbial concentrations of the produced water of production well Lu1039. The cores were sealed for 7 days at 40°C after nutrient injection. Water flooding was performed again until no further oil was observed in the eﬄuent to calculate oil displacement efficiency. The controls with only air and nutrients, or air and *B. subtilis*, were performed under the same conditions to provide information on the background levels.

**Table 2:** Core-flooding test showing the application potential of an exogenous lipopeptide-producing *Bacillus subtilis* M15-10-1 in assisting enhancement of oil recovery based on nutrient stimulation.

Tested project	Pore volume (ml)	Oil saturation (ml)	Water permeability (μm^2^)	Oil displacement efficiency (%)		
				First water flooding	Successive water flooding	oil displacement efficiency
*Bacillus subtilis*	59.5	48.5	2.06	45.65	48.27	2.62
Nutrients	61.6	49.2	2.15	46.25	53.84	7.59
Nutrients + *Bacillus subtilis*^a^	60.3	46.9	1.97	46.12	62.83	16.71
Nutrients + *Bacillus subtilis*^b^	62.1	48.9	2.09	47.58	58.54	10.96

*The nutrients consisted of 0.2% (NH_4_)_2_HPO_4_, 0.4% NaNO_3_, and 0.2% corn steep powder.*

*The Bacillus subtilis inoculation quantity was 0.5%, which was determined according to the microbial concentration of the produced water of the reservoir.*

^a^*Fermentation broth of Bacillus subtilis M15-10-1.*

^b^*Bacillus subtilis agent that was suspended with BSM.*

### Nucleotide Sequence Accession Number

The raw reads obtained in this study were deposited in the NCBI Nucleotide Archive database under project identification number PRJNA269199 (http://www.ncbi.nlm.nih.gov/bioproject/PRJNA269199).

## Results

### Metabolic Characteristics of *Bacillus subtilis* M15-10-1

**FIGURE 2:**
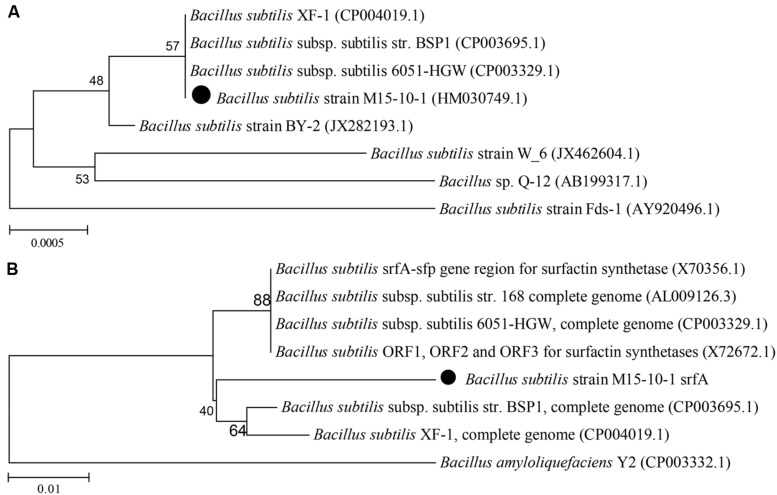
**Phylogenetic relationship based on **(A)** the 16S rDNA gene and **(B)***srfA* gene sequences between the *Bacillus subtilis* M15-10-1 strain and related species**.

**FIGURE 3:**
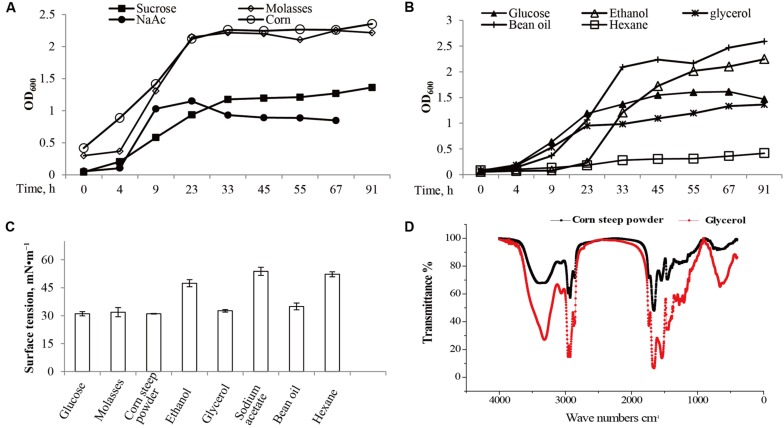
**Microbial growth and surfactant production of *Bacillus subtilis* M15-10-1 when grown with diverse carbon sources.** The growth curves are measured by **(A,B)** optical density (OD_600_), **(C)** the pH and surface tension of fermentation broth, and **(D)** the Fourier transform infrared spectrometry (FTIR) spectra of surfactants.

The strain M15-10-1 showed highest 16S rDNA sequence similarity with *B. subtilis* (**Figure 2A**). Detection of a surfactant-producing gene revealed that the M15-10-1 strain has the lipopeptide-producing gene *srfA*, suggesting that this strain may produce lipopeptides (**Figure 2B**). The dynamic growth curves indicated that the strain reached the exponential phase in 4–5 h, and rapidly entered the stationary phase within a short time when grown with diverse agro-industrial substrates as carbon sources (**Figures 3A,B**). As a result of biosurfactant synthesis, the surface tension of the cultures grown on glycerol, glucose, molasses, corn steep powder, and vegetable oil was reduced to approximately 30 mN⋅m^–1^ (**Figure 3C**). The surfactant production by the strain was approximately 300–500 mg L^–1^. The results indicated that various inexpensive nutrients could be used as substrates for M15-10-1 to produce surfactants. The lipopeptide-producing gene *srfA* detected in this strain and the FTIR spectra of the surfactant together revealed that *B. subtilis* M15-10-1 produced lipopeptides (**Figure 3D**). In addition, the *B. subtilis* cannot degrade crude oil (**Table 2**).

### Inhibitory Effect of Strain M15-10-1 on Reservoir Microbial Populations

**FIGURE 4:**
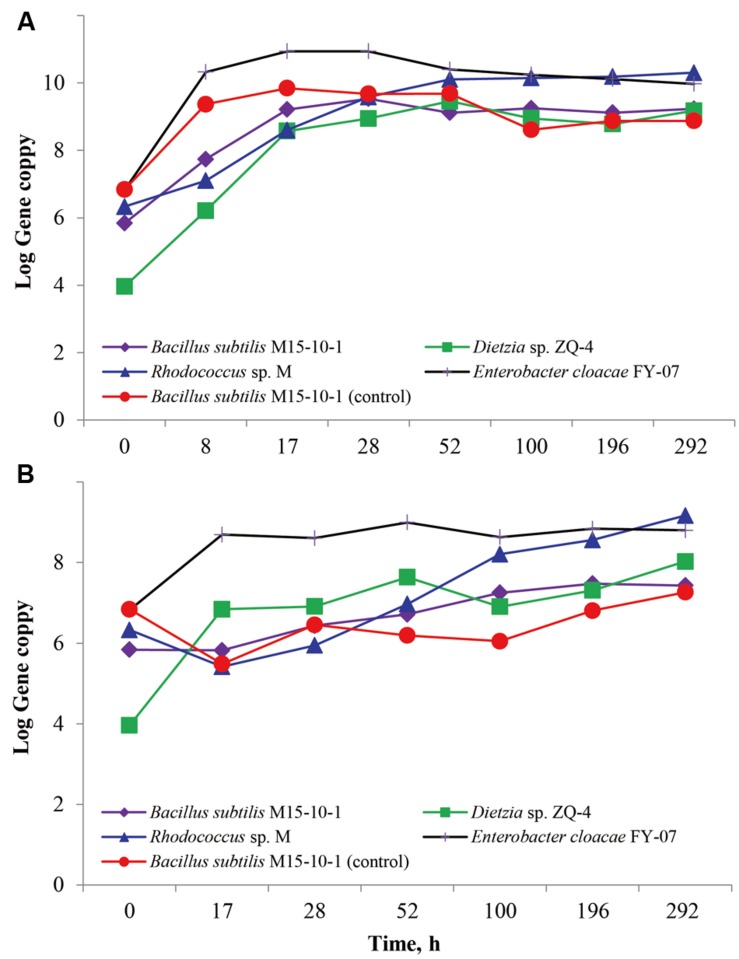
**Growth curves of *Bacillus subtilis* M15-10-1, *Dietzia* sp. ZQ-4, *Rhodococcus* sp. M, and *Enterobacter cloacae* FY-07 in an artificial microflora cultured on LB **(A)** and BSM **(B)** medium.** *Bacillus subtilis* M15-10-1 (control): Growth curves of *Bacillus subtilis* M15-10-1 in LB **(A)** and BSM **(B)** medium.

The inhibitory effect of the *B. subtilis* M15-10-1 on reservoir microbial populations in an artificial microflora was investigated using qPCR. As shown in **Figure 4A**, the *B. subtilis* had a similar growth curve whether cultured with other microbial populations or cultured alone in LB medium. The growth curves also indicated that the *B. subtilis* did not exert an observable inhibitory effect on the *Dietzia* sp. ZQ-4, *Rhodococcus* sp. M, and *E. cloacae* FY-07. Although the *B. subtilis* and *E. cloacae* cannot grow with oil as the carbon source, the number of the two species increased when cultured with the oil-degraders *Dietzia* sp. ZQ-4 and *Rhodococcus* sp. M in BSM crude oil medium (**Figure 4B**). This was clearly attributed to the growth of *Dietzia* and *Rhodococcus*, which are well-known for their oil-degradation ability and surfactant production.

### Effect of Strain M15-10-1 on Oil Emulsification and Microbial Growth During Nutrients Stimulation

**FIGURE 5:**
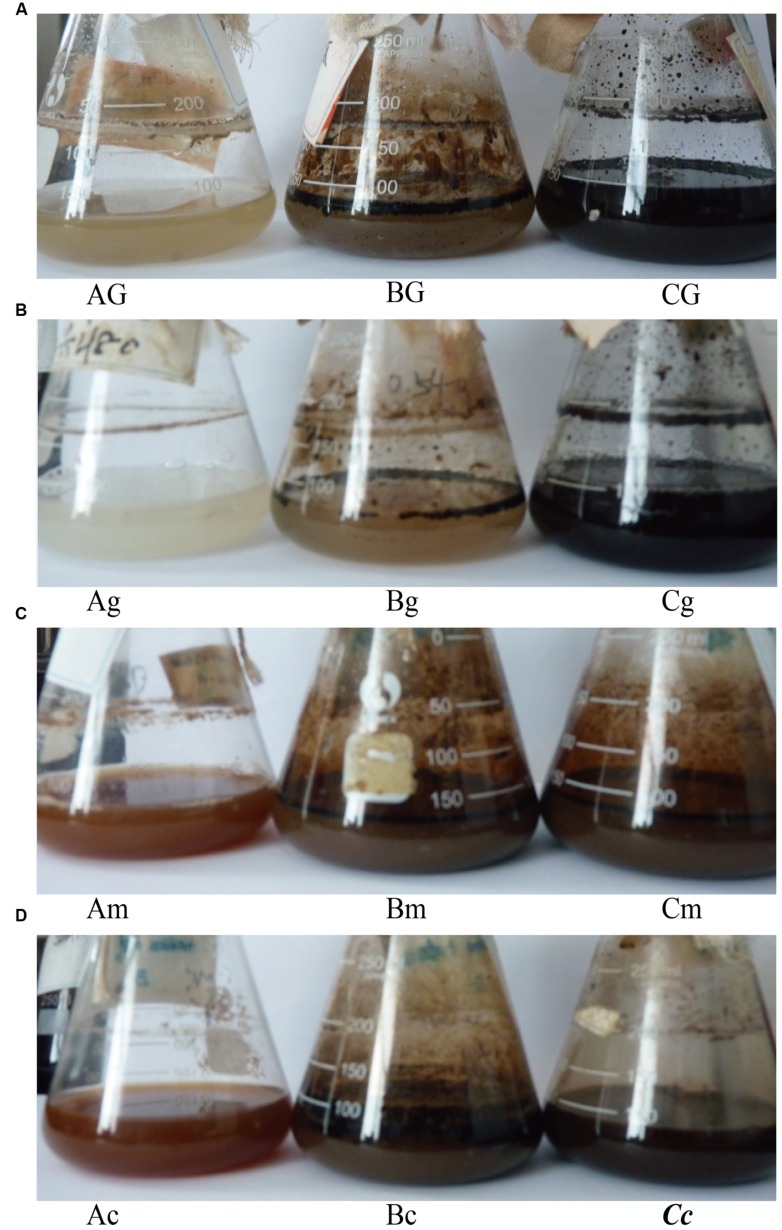
**Oil emulsification during stimulation with nutrients or in combination with *Bacillus subtilis* M15-10-1.** Different combinations are presented in images **(A–D)**. A: microcosm containing nutrients; B: microcosm containing nutrients and crude oil; C: microcosm containing nutrients, crude oil, and *Bacillus subtilis* M15-10-1; G: glucose; g: glycerol; m: molasses; and c: corn steep powder.

**FIGURE 6:**
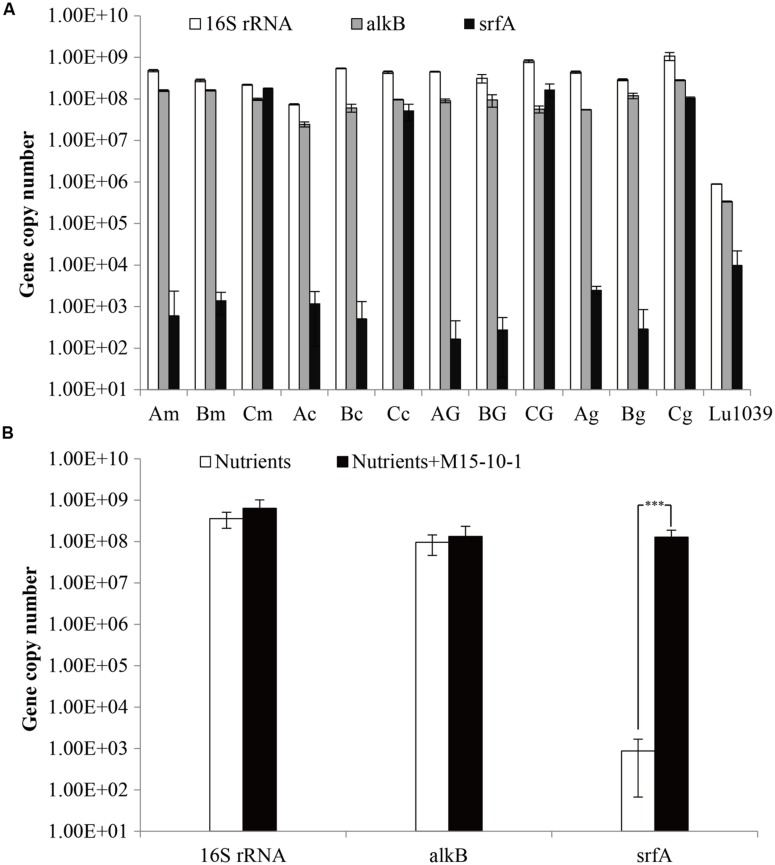
**Copy numbers **(A)** and average copy numbers **(B)** of 16S rRNA, *alkB* and *srf*A genes detected in the water samples stimulated by nutrients or in combination with *Bacillus subtilis* M15-10-1.** A: microcosm containing nutrients; B: microcosm containing nutrients and crude oil; C: microcosm containing nutrients, crude oil, and *Bacillus subtilis* M15-10-1; G: glucose; g: glycerol; m: molasses; and c: corn steep powder. ^∗∗∗^The abundance of the gene was significantly different among water samples stimulated by nutrients or in combination with *Bacillus subtilis* M15-10-1 (*P* < 0.0001).

Both glucose and glycerol stimulated the growth of indigenous microorganisms, but did not improve oil emulsification in the process of stimulation of reservoir microorganisms. The results indicated that neither glucose nor glycerol stimulated indigenous microorganisms to produce enough surface-active agents to emulsify crude oil during stimulation. In contrast, molasses and corn steep powder not only stimulated microbial growth, but also improved oil emulsification (**Figure 5**). However, it was questionable which factors resulted in oil emulsification during stimulation. We therefore investigated the microbial concentration and oil degradation before and after nutrient stimulation. The qPCR results indicated that, even though there was no crude oil in the microcosms, the number of *alkB* genes reached 10^7^–10^8^ copies mL^–1^. In the microcosms with glucose or glycerol, which could not stimulate reservoir microorganisms to emulsify crude oil, the number of *alkB* genes also reached 10^7^–10^8^ copies mL^–1^ (**Figure 6**). Unexpectedly, no oil degradation was clearly observed in these microcosms, with saturated hydrocarbons decreasing from 71.29 to 65.45% and 64.31% in the glucose and glycerol treatments, respectively (**Table 3**). These results suggest that the growth of hydrocarbon-degrading bacteria may not have a causal relationship with oil emulsification during stimulation, whereas the specific nutrient additions may be crucial for stimulating reservoir microorganisms to produce surfactants to emulsify oil.

**Table 3:** Relative quantities of saturated hydrocarbons, aromatic hydrocarbons, asphaltene, and non-hydrocarbons in crude oil before and after stimulation with nutrients or combination with *Bacillus subtilis* M15-10-1.

Experiments	Relative quantity of crude oil (%)			
	Saturated hydrocarbon	Aromatic hydrocarbon	Asphaltene	Non-hydrocarbon
Control	71.29	14.85	5.94	5.94
Control + M15-10-1	70.28	12.24	3.85	7.69
Glucose	65.45	16.97	8.48	5.45
Glucose + M15-10-1	58.89	22.22	2.96	12.59
Glycerol	64.31	18.73	2.12	9.19
Glycerol + M15-10-1	61.35	18.40	4.60	12.88
Molasses	55.38	18.88	9.38	15.13
Molasses + M15-10-1	54.69	22.92	3.65	14.58
Corn steep powder	51.52	24.24	13.13	9.09
Corn steep powder + M15-10-1	48.28	18.23	12.81	20.20

**FIGURE 7:**
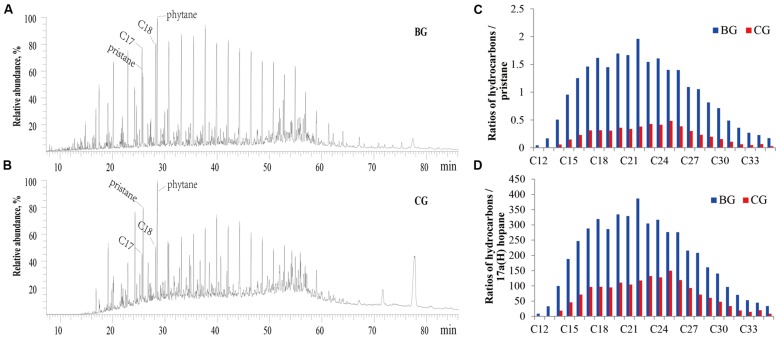
**Gas chromatography profile of total hydrocarbon **(A,B)** and the relative ratios of different chain-length hydrocarbons to pristine or 17α(H)-hopane in crude oil **(C,D)** during stimulation with nutrients or in combination with *Bacillus subtilis* M15-10-1.** BG: microcosm containing glucose; CG: microcosm containing glucose and *Bacillus subtilis* M15-10-1.

Although glucose and glycerol could not stimulate reservoir microorganisms to emulsify crude oil, the added *B. subtilis* M15-10-1 (the inoculation quantity was 0.5%, which was determined according to the indigenous microbial concentration) rapidly improved oil emulsification during stimulation. Interestingly, the exogenous *B. subtilis* M15-10-1 also accelerated oil degradation, with saturated hydrocarbons decreasing from 71.29 to 58.89% and 61.35% in the glucose and glycerol treatments, respectively (**Figure 7** and **Table 3**). Generally, pristane and phytane are not depleted by initial to moderate biodegradation, while 17α(H)-hopane is used as a conservative marker to calculate biodegradation rates (%) for saturated hydrocarbons. In this study, there was an obvious decrease in hydrocarbons and pristane and hydrocarbons and 17α(H)-hopane in microcosms with M15-10-1 compared with the control with only nutrient additions (**Figures 7C,D**). Furthermore, the ratios of hydrocarbons (C12–C35) all decreased in both microcosms with nutrient additions and strain M15-10-1. It is reasonable to deduce that the added *B. subtilis* M15-10-1 produced surfactants, which emulsified crude oil into droplets that further enhanced the growth of indigenous hydrocarbon-degrading bacteria with crude oil as the carbon source.

### Response of Reservoir Microbial Community to the *Bacillus subtilis* During Stimulation

**FIGURE 8:**
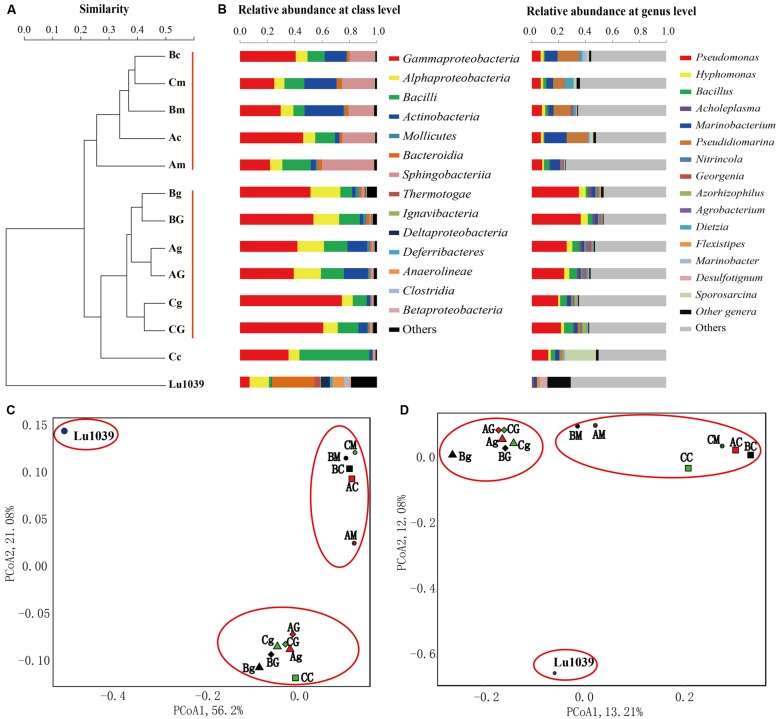
**Clustering **(A)**, histogram **(B)**, weighted Unifrac PCoA **(C)**, and unweighted Unifrac PCoA **(D)** analysis of the microbial communities in water samples stimulated by nutrients or in combination with *Bacillus subtilis* M15-10-1.** A: microcosm containing nutrients; B: microcosm containing nutrients and crude oil; C: microcosm containing nutrients, crude oil, and *Bacillus subtilis* M15-10-1; G: glucose; g: glycerol; m: molasses; and c: corn steep powder.

To elucidate the ecological influence of exogenous *B. subtilis* M15-10-1 on indigenous microbial communities, microbial abundance and community structures in the microcosms with nutrients, or nutrients and exogenous *B. subtilis* under limited air supply conditions, were investigated by qPCR and high-throughput sequencing. Compared with the microcosms stimulated with nutrients, the numbers of *srfA* genes reached 10^8^ copies mL^–1^ in the microcosms with nutrients and *B. subtilis* M15-10-1 (**Figures 6A,B**). The results indicated that the added *B. subtilis* adapted to and grew in the new environments. However, it was questionable whether the growth of the *B. subtilis* exerted an inhibitory effect on the indigenous microorganisms. To explore the issue, community diversity and composition of each microcosm were analyzed. There was no obvious difference in the numbers of 16S RNA, *alkB*, and Shannon diversity index in microcosms with nutrients and exogenous *B. subtilis* compared with the microcosms with only nutrient additions (**Figure 6B** and Supplementary Figure S1). In microcosms with nutrients and the *B. subtilis*, the genus *Bacillus* only accounted for 2.2–6.6% of the communities (**Figure 8B**). The result is coincident with the quantification of the lipopeptide-producing gene *srfA*. These data indicated that the added exogenous *B. subtilis* and the produced surfactant did not exert an obvious inhibitory effect on the growth of the indigenous microorganisms during stimulation.

The phylogenetic variation between the stimulated microhabitats was further measured by UniFrac distances. Histograms, hierarchical clustering, and PCoA biplots were used to interpret the relative similarity of the microbial communities (**Figure 8**). The results indicated that the nutrient types exerted stronger influences on community structures than the added exogenous *B. subtilis*. As shown in **Figure 8**, the communities from microcosms with glucose and glycerol were classified as a group, while those of microcosms with molasses and corn steep powder were classified as another group (**Figures 8C,D**). Additionally, the microcosms with the *B. subtilis*, in particular, those stimulated by glucose and glycerol, have similar community structures to each other rather than those with only nutrient additions. The results may be related to the following facts: (1) compared with molasses and corn steep powder, glucose and glycerol have a single nutritional component, which may be only fit for the stimulation of several microbial populations, e.g., *Pseudomonas*; (2) the growth of the exogenous *B. subtilis* made the indigenous communities have a more similar community structure.

### Core Oil-Displacement Test

The core-flooding test was designed to simulate the MEOR process. The results of the core-flooding tests are shown in **Table 2**. The results indicated that the brines with nutrients (0.2% (NH_4_)_2_HPO_4_, 0.4% NaNO_3_, and 0.2% corn steep powder) and 0.5% fermentation broth of *B. subtilis* M15-10-1 increased the oil displacement efficiency by 16.71%, while it increased by 10.96% with nutrients and 0.5% *B. subtilis* agent that was suspended with BSM. In contrast, it only increased by 7.59% with nutrient injection only, and by 2.62% with *B. subtilis* agent injection only.

## Discussion

Indigenous microbial enhanced oil recovery (IMEOR) is driven by the synthetic action of reservoir microorganisms and their metabolites, including surfactants, organic acids, CO_2_, and CH_4_ (Youssef et al., 2009; Voordouw, 2011). In IMEOR treatment, surfactant production is the first stage of nutrient stimulation (**Figure 1**), and is thought to be the most critical stage (Li et al., 2014). However, in most cases, it is difficult for reservoir microorganisms to produce enough surfactants to emulsify oil under limited air supply conditions. To improve this process, an exogenous lipopeptide-producing *B. subtilis* was used to facilitate the IMEOR process. The microbial processes and driving mechanisms were investigated by analyzing the changes in oil properties and the response of the indigenous microbial community when stimulated with nutrients or the exogenous *B. subtilis*. The results provided useful information to increase our understanding of the microbial processes occurring in the process of exogenous surfactant-producers strengthening IMEOR and for developing an efficient MEOR technique.

Along with the development of the MEOR technique, a large number of microbial populations, including commonly reported *Pseudomonas*, *Rhodococcus*, *Bacillus*, and *Dietzia*, have been detected and isolated from reservoir environments using culture-based methods and culture-independent techniques (Simpson et al., 2011; Xia et al., 2012; Wang et al., 2013, 2014a; Li et al., 2014). Most of these populations can produce surfactants when grown with crude oil as the carbon source. These properties have resulted in them receiving increasing attention from both researchers and oil companies. To date, *Pseudomonas* and *B. subtilis* have been widely used in the petroleum industry to aid oil recovery due to their ability to produce surfactants with highly desirable properties for oil recovery (Simpson et al., 2011; You et al., 2015). However, their oil-degrading ability makes it difficult to determine the influence of these populations when used to strengthen the IMEOR process. The *B. subtilis* M15-10-1 can grow and produce lipopeptides on various cheap carbon sources, except for crude oil, making it an ideal candidate for analyzing the role of exogenous microbial populations in the IMEOR process.

Water-flooded oil reservoirs contain diverse microbial populations. High-throughput sequencing has revealed that abundant potential surfactant-producing bacteria, in particular *Bacillus*, *Pseudomonas*, and *Dietzia*, inhabit the Lu water-flooded reservoir (Gao et al., 2015b). Most species of these populations have been isolated and demonstrated to be able to produce surfactants on multiple hydrocarbons and carbohydrates, e.g., crude oil, sugar, and grease (Xia et al., 2012; Gudina et al., 2013; Wang et al., 2013). Unexpectedly, it is hard to stimulate these microorganisms to emulsify crude oil when adding nutrients into the formation brines. The present study revealed that different kinds of nutrient compounds could stimulate the growth of indigenous microorganisms, but not all the nutrients improved oil emulsification in the stimulation process (**Figure 5**). This elucidated the reason why specific nutrient compounds were selected and used in different reservoirs (Belyaev et al., 1998; Liu et al., 2005; Bao et al., 2009; Li et al., 2014; Halim et al., 2015). However, it is easy to envisage that once these surfactant producers are domesticated in the laboratory, they may produce sufficient surfactants to emulsify crude oil when grown on multiple carbon sources. In the present study, glucose and glycerol did not stimulate the reservoir microorganisms to emulsify crude oil, but the added *B. subtilis* M15-10-1 rapidly improved oil emulsification. This phenomenon suggests that the exogenous *B. subtilis* can improve oil emulsification in the IMEOR process, in particular for the reservoirs where different kinds of nutrients cannot effectively improve oil recovery. Importantly, the exogenous *B. subtilis* also accelerated oil biodegradation, indicating that the emulsified oil may further enhance the growth of indigenous hydrocarbon-degrading bacteria with crude oil as the carbon source during stimulation (**Figure 7**).

Whether an exogenous MEOR treatment or exogenous population facilitates the IMEOR process, the added surfactant-producers must be able to grow in the presence of competing indigenous microorganisms. In an artificial microflora, we found that the exogenous *B. subtilis* M15-10-1 could live together with the ubiquitous reservoir microbial populations. It seems that the *B. subtilis* has no obvious inhibitory effect on these microorganisms. In addition, although the *B. subtilis* cannot grow with oil as the carbon source, the growth of the oil-degraders *Dietzia* and *Rhodococcus* improved the growth of the *B. subtilis* when cultured with crude oil as the sole carbon source (**Figure 4**). We further investigated the effect of the *B. subtilis* on the indigenous microbial community. The qPCR results indicated that the added *B. subtilis* adapted to and grew in concert with the indigenous microbial populations (**Figure 6**). There was also no obvious difference in the numbers of 16S RNA, *alkB*, and alpha diversity in microcosms with exogenous *B. subtilis* compared with the microcosms with only nutrient additions (**Figure 6B** and Supplementary Figure S1).

Petroleum reservoirs represent special environments underground with low permeability. Owing to the sieve effect on microbial cells when injected fluid passes through a subsurface formation, the injected exogenous microorganisms may not migrate into the reservoir formation (Youssef et al., 2009; Gao et al., 2015a; Ren et al., 2015). Youssef et al. (2007) injected *B. licheniformis*, *B. subtilis* subsp. *Spizizenii*, and nutrients into two Viola formation wells, and improved oil recovery. They also pointed out that the injected exogenous microorganisms may only affect a radius of 2–4 m from the well in a follow-up study (Simpson et al., 2011). In this case, the injected exogenous bacteria will mainly grow and colonize the near-wellbore area. However, it may be suitable for an IMEOR treatment. When exogenous surfactant producers are injected into reservoir strata by a water injection well, most of these microorganisms may accumulate in the near-wellbore area of water injection well. The immobilized microorganisms may serve as a “seed bank.” Therefore, once nutrients are injected through a water injection well, the “seed bank” will transport the surfactant producers into reservoirs continuously. Then, the surfactant producers will produce surfactants to strengthen the IMEOR process.

The core-flooding test is an economic model, and can simulate the oil-recovery operations usually conducted in reservoirs (Gudina et al., 2013). It was used to evaluate the application potential of the exogenous surfactant-producing *B. subtilis* in facilitating the IMEOR process. Previous investigations have shown that the stimulation of indigenous microorganisms enhances oil recovery by 3.7–9.14 % (Bao et al., 2009; Dong et al., 2015), and by 4.89–24 % in core-flooding tests with surfactant-producing bacteria and their metabolites (She et al., 2011; Castorena-Cortes et al., 2012; Xia et al., 2012; Gudina et al., 2013; Al-Wahaibi et al., 2014; Arora et al., 2014; Song et al., 2015). In this study, the brines with nutrients and fermentation broth of *B. subtilis* M15-10-1 increased the oil displacement efficiency by 16.71%, which is higher than the 7.59% with nutrient injection only. However, the *B. subtilis* can only grow and produce surfactants in the presence of oxygen, otherwise higher oil displacement efficiency may be realized. In this instance, facultative anaerobic surfactant-producing bacteria, such as *Geobacillus stearothermophilus*, may be an alternative candidate.

In summary, this study investigated the microbial processes and driving mechanisms in an exogenous *B. subtilis*-facilitated IMEOR process. The exogenous *B. subtilis* could live together with reservoir microbial populations, and did not show an obvious inhibitory effect on the indigenous microorganisms. The *B. subtilis* facilitated the IMEOR process by improving oil emulsification and accelerating microbial growth with oil as the carbon source. Core-flooding tests showed the great application potential for enhancing oil recovery by combining exogenous and indigenous microbial flooding technology. This oil recovery technique has obvious advantages: (1) the injected microbial populations may accumulate and form a “seed bank” in the near-wellbore area of water injection well; and (2) this technique is especially suited to reservoirs where IMEOR treatment cannot effectively improve oil recovery.

## Author Contributions

PG, GL, and TM conceived and proposed the idea. PG, Yan Li, Yanshu Li, HT, YW, and JZ carried out the experiments and conducted data analysis. PG and TM drafted the manuscript. All authors have read and approved the final manuscript.

## Conflict of Interest Statement

The authors declare that the research was conducted in the absence of any commercial or financial relationships that could be construed as a potential conflict of interest.
