# Supplementary material for: An Exogenous Surfactant-Producing Bacillus subtilis Facilitates Indigenous Microbial Enhanced Oil Recovery
Source: Front Microbiol. 2016 Feb 18;7:186. doi: 10.3389/fmicb.2016.00186 (PMC4757698; doi:10.3389/fmicb.2016.00186)
Supplement: Supplementary file 1 [file Presentation_1.PDF]

Supplementary material

An exogenous surfactant-producing *Bacillus subtilis* facilitates indigenous microbial enhanced oil recovery

Running title: *Bacillus subtilis* facilitates oil recovery

Peike Gao[1], Guoqiang Li[1], Yanshu Li[1], Yan Li[1], Huimei Tian[1], Yansen Wang[1], Jiefang Zhou[1] and Ting Ma[1]*

[1] Key Laboratory of Molecular Microbiology and Technology, Ministry of Education, College of Life Sciences, Nankai University, Tianjin 300071, P. R. China.

Correspondence:

Ting Ma

College of Life Sciences, Nankai University, Tianjin 300071, P.R. China

tingma@nankai.edu.cn

Conflict of Interest Statement: The authors declare that the research was conducted in the absence of any commercial or financial relationships that could be construed as a potential conflict of interest.

Tabale S1 Physicochemical characteristic of the water samples obtained from the Lu water-flooding reservoir

Nutrient elements, mg/L	Lu1039	Lu3073	Lu3084	Lu2180	Lu3095	Average
Total nitrogen	11.5	12.7	15.1	10.6	11.6	12.3
Total phosphorus	19.1	18.1	20.2	17.5	19.5	18.88
SO_4^{2-}	14.04	23.13	116.2	4.86	8.86	33.41
HCO_3^-	434.09	846.29	356	464	511.42	522.36
Cl^-	6125	5160	5640	5820	5850	5719
Na^+	4759	4275	4460	4516	4448	4491.6
K^+	44.1	33.77	64.9	49.35	38.96	46.22
Ca^{2+}	281.9	181.6	191.3	284.7	216.4	231.18
Mg^{2+}	32.07	26.03	21.7	31.55	28.83	28.04

Detected limit was 0.1mg/L.

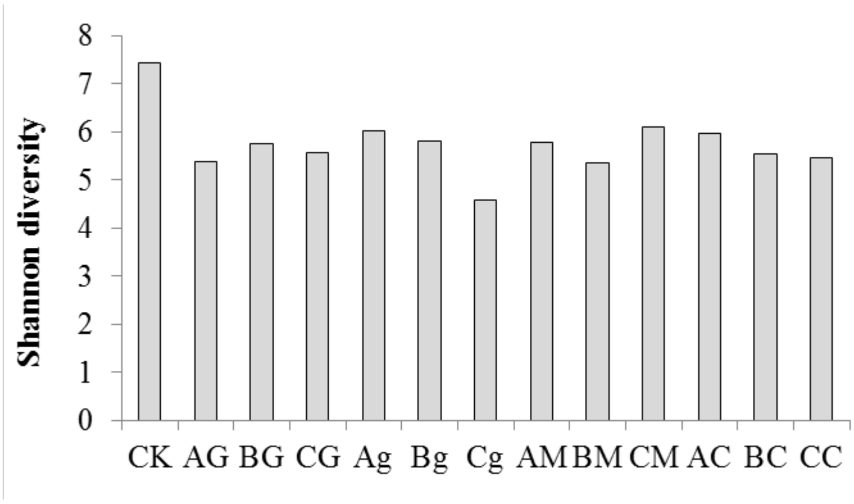

Fig. S1 Shannon diversity index of microbial communities in the water samples stimulated by nutrients or in combination with *Bacillus subtilis* M15-10-1. Ck represents the control water sample Lu1039; A: microcosm containing nutrients; B: microcosm containing nutrients and crude oil; C: microcosm containing nutrients, crude oil, and *Bacillus subtilis* M15-10-1; G: glucose; g: glycerol; m: molasses; and c: corn steep powder.
